# A timely review of a key aspect of motor imagery: a commentary on Guillot et al. (2012)

**DOI:** 10.3389/fnhum.2013.00761

**Published:** 2013-11-08

**Authors:** Dave Smith, Caroline Wakefield

**Affiliations:** ^1^Department for Exercise and Sport, Manchester Metropolitan UniversityCrewe, UK; ^2^School of Health Sciences, Liverpool Hope UniversityLiverpool, UK

**Keywords:** mental practice, mental chronometry, imagery timing, bio-informational theory, rhythm

## Abstract

The timing of motor imagery has recently received attention from a number of researchers, culminating in a comprehensive review by Guillot and colleagues. This paper aims to further explore this issue, building upon the said review to suggest a number of other important timing-related issues. Specifically, we consider the possible role of bio-informational theory (Lang, 1979, 1985) and the recent proposal of “behavioral matching” in conjunction with the PETTLEP model (Holmes and Collins, 2001) of motor imagery. Furthermore, we explore the possibility that timing has important implications for motivational aspects of imagery. We then discuss the potential role of rhythm, an important but often overlooked aspect of skilled motor performance, and its links to the timing issue. Finally, we conclude by offering suggestions for future imagery timing research to examine this relatively under-researched area of imagery.

## Introduction

Imagery is one of the most popular psychological techniques used in sports skill learning. However, despite growing knowledge of how skills are best learned, there is still some lack of agreement regarding the most effective ways to implement imagery interventions. One issue that has received a great deal of recent research scrutiny is the speed at which the imagery should be conducted to have the greatest performance benefits. Imagery can be performed in real time, or there can be a divergence between the time taken to perform a movement and to mentally simulate it. This may be deliberate or because an individual is not capable of producing a vivid image in real time. For example, individuals may perform slow motion imagery deliberately when developing a skill, to enable them to focus more on key aspects of that skill than would be possible when performing real-time imagery (O and Hall, 2009). Also, stroke rehabilitation patients may perform slow imagery as following a stroke motor cognition slows down (González et al., 2005). Alternatively, an athlete may, when mentally simulating a skill, imagine him or herself to perform the skill more quickly than he or she currently does, as faster performance is desirable ( e.g., in running a race). A recent review by Guillot et al. (2012) addressed many of the associated issues and provided a clear and comprehensive examination of work in this area. In order to respond to this, we would like to add our own suggestions for future research and raise issues that we believe could further develop understanding of this component of imagery research.

## Bio-informational theory

Researchers in sport psychology have long been intrigued by the possible applications of Lang’s (1979, 1985) bio-informational theory to motor imagery (see, for example, Hale, 1982, 1994). This theory was proposed to explain the effects of imagery interventions in treating emotional disorders, but the theory also seems to apply well to the imagery of motor skills. Indeed, its tenets have been well-supported in the sport psychology literature (Bakker et al., 1996; Smith et al., 2001; Slade et al., 2002; Smith and Collins, 2004; Wilson et al., 2010). Lang posited that all knowledge is represented in memory as units of information regarding objects, relationships and events. These units of information are termed propositions, of which there are three fundamental categories represented in memory: stimulus, response and meaning propositions. Stimulus propositions are the descriptive referents relating to the external environment. Response propositions describe the responses of the individual to the stimuli in the scene, such as motor activity and autonomic changes. Meaning propositions are analytical and interpretative, adding components of information not available from the stimuli in the situation. They define the significance of events and the consequences of action.

According to Lang (1985), the processing of response propositions accesses the memory representation for the imaged movement, and thus leads to physiological responses in relevant muscles and organs. Also, meaning propositions must be processed to fully access the memory of the action. It is the accessing, and subsequent strengthening, of the memory representation that is hypothesized to enhance performance. We might expect that imagery performed at the same speed as the task is actually performed would be more meaningful to the performer than slower or faster imagery, having stronger meaning propositional content. According to bio-informational theory such greater meaningfulness should translate into more effective imagery, but such a suggestion has yet to be tested from a Langian perspective. In addition, the timing issue has important implications for response propositions and the kinesthesis that results from the processing of these. Specifically, the kinesthetic sensations accompanying a movement are partially dictated by the timing of that movement, as changes in the timing will lead to changes in the pattern of muscle activation that produces the kinesthetic sensations being experienced. This is because movement kinematics change as movement speed changes (for example, Brindle et al., 2006), therefore we hypothesize that real time imagery will be more likely to be associated with realistic, meaningful kinesthesis than will slow motion or fast imagery. However, this has yet to be tested empirically, and thus examinations of the effects of imagery timing on the propositional content of the imagery experience (specifically response and meaning propositions) would be very welcome additions to the imagery literature.

## Behavioral matching

The development of the PETTLEP model (Physical, Environment, Task, Timing, Learning, Emotion, Perspective; Holmes and Collins, 2001) provided some practical guidelines for imagery interventions. The model was based on findings from neuroscience (Jeannerod, 1997) and cognitive psychology (see Lang’s work cited in the preceding section). It centered on the premise that a “functional equivalence” exists between imagery and execution of a task. However, a review by Wakefield et al. (2013) further explored this issue and concluded that behavioral matching may be a more appropriate term for the interventions used in most published research on this topic, as the similarity described in these studies is more at a behavioral level, and merely reflects and implies neural equivalence. As such, they recommended that the behavioral aspects of PETTLEP imagery be matched as closely as possible to actual execution of a task.

Timing is one such component of the PETTLEP model and, as such, if behavioral matching is to occur then imagery interventions should be conducted in real time, appropriate to the learning stage of the performer. O and Hall (2009) tested the intentional use of imagery at different speeds, reporting that slow motion imagery was used more frequently when learning a new skill. Timing has also been shown to be adversely affected when imagery is performed in a relaxed condition (Louis et al., 2011). This further supports the notion that imagery should be matched to the behavioral characteristics of physical performance. However, skilled performers can intrinsically control the speed of their imagery (Munroe et al., 2000; Morris et al., 2005). This is interesting in the context of PETTLEP as Holmes and Collins (2001) suggested there may be differences in the imagery experience, and the meaningfulness of it, dependent upon the stage of learning. Despite the mixed findings regarding the relative efficacy of different imagery timings, further research on this topic is important to establish the optimal imagery conditions for enhanced performance.

Recent work in our own laboratories has focused on manipulation of imagery speed within the framework of the PETTLEP model. The work has assessed the impact on performance of sport and fitness-based tasks, with imagery conducted at real time, increased speed and slow motion using video-controlled timing (i.e., using action observation concurrently to imagery, with participants instructed to mentally simulate the movement whilst watching a first-person perspective video of it). Preliminary results have generally revealed a positive impact on performance regardless of imagery speed. However, the real time and slow motion groups have shown the largest performance increases. Therefore, this evidence does not unequivocally support the idea that real time imagery should generally be used to facilitate the behavioral matching process. Indeed, depending on the stage of learning of the performer or their particular performance goals, slow motion may be equally effective, as slow motion imagery has been shown to have advantages for athletes trying to correct a bad habit (Syer and Connolly, 1984). Specifically, slow motion imagery will enable the athlete to see and feel faults in technique in a way that might be impossible with real time imagery, particularly with skills that are performed in a very short space of time, such as specific parts of a gymnastics move or a dive. In such cases the movement would be over so quickly that it would be difficult for the athlete to focus in any detail on specific parts of it whilst imaging in real time. Slow motion imagery, on the other hand, may enable the athlete to explore different parts of the movement more effectively. Thus, the efficacy of real-time versus slow motion imagery may be achieved through slightly different mechanisms, with real-time imagery providing a very meaning-laden and behaviorally-matched imagery experience to enable realistic mental practice (cf. the PETTLEP model, Wakefield et al., 2013) and slow motion imagery enabling an explicit analysis of technique, enabling performance enhancement through modifications made in response to such analysis.

## Motivational aspects

Guillot et al. (2012) focused their attention on the cognitive specific function of motor imagery (i.e., the use of imagery to mentally simulate movements), stating that there is no reason to presume that imagery speed might influence motivational imagery’s effectiveness. However, cognitive specific imagery may also produce motivational effects, and imagery speed may well be a confounding factor in such effects, particularly in activities where speed is a crucial element of performance. It seems reasonable to presume that imaging such activities faster than they can be carried out at present (such as a sprinter imagining performing a personal best time) may well have strong motivational impact. Conversely, imaging such activities more slowly than would normally be performed (such as a triple jumper imaging performing their run-up in slow motion to help correct a technical fault) would be less likely to have a motivational impact, though the imagery may still serve a very useful purpose. More research is therefore needed to examine the effects of different imagery speeds on the motivational impact of cognitive-specific imagery.

## Rhythmicity

A further issue relating to the timing of imagery that could benefit from more research is the rhythmicity of the action. Many, if not all, sports skills can be considered rhythmic in nature (Gallahue and Donnelly, 2003), and rhythm, or “temporal invariance of movement components” (MacPherson and Collins, 2009, p.S49), is a crucial aspect of many sport skills. Thus, whereas timing in imagery corresponds to the duration or speed of a global task, rhythmicity relates to the relative timing of different parts of a task, such as when a series of co-ordinated actions are performed. Links have been shown between rhythmicity and performance of a number of sports including gymnastics (Pica, 1998), golf (Kim et al., 2011a), dance (Laurence, 2000), fencing (Borysiuk and Waskiewicz, 2008), swimming (Zachopoulou et al., 2000) and tennis (Sogut et al., 2012). Rhythm, like imagery, is an important component in ensuring effective preparation for competition (MacPherson and Collins, 2009). Research has shown that as skill level improves, there is a decrease in the degree to which the movement sequence varies (Rose and Christina, 2006). Thus, it could be argued that increased rhythm is achieved when learning progresses and stable rhythmic structures are apparent in mature motor skill patterns. However, research has shown increased temporal variability, thus reduced rhythm, with increasing age (Kim et al., 2011b). The rhythm of the action to be imaged may, therefore, have an impact on the optimal imagery conditions, and should be considered when designing interventions. Also, the degree to which rhythm is a necessary component of a particular skill may influence the effect of varied timing of interventions on that same skill. MacPherson and Collins (2009) argue that promoting mechanisms controlling the consistency of timing and rhythm is a worthy endeavor in the field of sport psychology.

Furthermore, Calmels et al. (2006) revealed that, whilst total time was comparable between imagery and execution, differences were apparent in the relative timing of the components. Therefore, focused imagery and observation interventions may not assist in ensuring and maintaining the rhythmical aspects of the components of sports skills: an area that warrants further research. The influence of factors such as imagery modality, agency and perspective on relative timing of movement components during imagery may be particularly worthwhile, to determine whether behavioral matching of imagery and movement execution may be more effectively achieved when such variables are manipulated in particular ways. For example, research (White and Hardy, 1995; Hardy and Callow, 1999) has found that third-person visual perspective imagery is more effective at enhancing the performance of form-based skills, such as gymnastic tasks, than the first person visual perspective. Given that rhythm is often a crucial component of such skills, and that the third person perspective provides a model of performance from which key aspects of the movement can be extracted, including rhythm, we hypothesize that external visual imagery may be more effective in reinforcing the desired rhythm than internal visual imagery. This is especially likely if the external perspective imagery is accompanied by kinesthesis, as imaging the feel of the movement may also help the imager mentally simulate the desired rhythm, which will no doubt be associated with particular kinesthetic sensations. The testing of such hypotheses would be a very useful addition to the imagery literature.

## Conclusion

In conclusion, we have highlighted some further areas that may impact imagery timing and the efficacy of different intervention speeds. Each of these areas would benefit from further research. Indeed, simply from a practical point of view, completing imagery at an increased speed enables more “sets” to be completed within a given intervention period. Additionally, this would also benefit performers in situations where there is a lack of available time (i.e., between points in a match). However, an increased speed of imagery could well have a detrimental effect on the quality of the imagery, though this is an issue that remains to be investigated. It is therefore important to fully understand the benefits and drawbacks of the varying timings of imagery, in order that the correct intervention can be matched to the age, performance level and sport of the individual. As such, we recommend future research should focus on the potential motivational effects of imagery timing, the link to meaning and the potential overlap with producing rhythmical action.

## conflict of interest statement

The authors declare that the research was conducted in the absence of any commercial or financial relationships that could be construed as a potential conflict of interest.
